# Bioengineered Extracellular Vesicle Hydrogel Modulating Inflammatory Microenvironment for Wound Management

**DOI:** 10.3390/ijms252313093

**Published:** 2024-12-05

**Authors:** Yunfei Mu, Liwen Ma, Jia Yao, Dan Luo, Xianguang Ding

**Affiliations:** 1 State Key Laboratory of Organic Electronics and Information Displays, Jiangsu Key Laboratory of Smart Biomaterials and Theranostic Technology, Nanjing University of Posts and Telecommunications, Nanjing 210023, China; 1223066206@njupt.edu.cn (Y.M.); 1023233416@njupt.edu.cn (J.Y.); 2 The First College of Clinical Medicine, Nanjing Medical University, Nanjing 211100, China

**Keywords:** extracellular vesicles, bioactive hydrogel, tissue healing

## Abstract

Chronic wounds, frequently arising from conditions like diabetes, trauma, or chronic inflammation, represent a significant medical challenge due to persistent inflammation, heightened infection risk, and limited treatment solutions. This study presents a novel bioengineered approach to promote tissue repair and improve the healing environment. We developed a bioactive hydrogel patch, encapsulated zeolitic imidazolate framework-8 (ZIF-8) into extracellular vesicles (EVs) derived from anti-inflammatory M2 macrophages, and synthesized ZIF@EV, then embedded it in the sodium alginate matrix. This hydrogel structure enables the controlled release of therapeutic agents directly into the wound site, where it stimulates endothelial cell proliferation and promotes new blood vessel formation. These processes are key components of effective tissue regeneration. Crucially, the EV-infused patch influences the immune response by polarizing macrophages towards an M2 phenotype, shifting the wound environment from inflammation toward regenerative healing. When applied in a murine model of chronic wounds, the EV hydrogel patch demonstrated notable improvements in healing speed, quality, and tissue integration compared to traditional approaches such as growth factor therapies and foam dressings. These promising findings suggest that this bioactive hydrogel patch could serve as a versatile, practical solution for chronic wound management, providing an adaptable platform that addresses both the biological and logistical needs of wound care in clinical settings.

## 1. Introduction

Wounds are a common issue in daily life, ranging from minor skin abrasions and scratches to larger surgical incisions. Regardless of their size, wounds can have a profound impact on quality of life [1,2]. Therefore, wound repair is a crucial topic in the field of medicine, involving several key factors in the tissue microenvironment [3]. The inflammatory state of the tissue assumes a central role in the process of wound repair. When an injury occurs, the body rapidly triggers an inflammatory response, which constitutes a natural physiological mechanism. This response is geared towards eliminating bacteria and debris from the injured region while concurrently signaling the commencement of the repair process [4,5]. However, an excessive or inadequate inflammatory response can negatively affect wound healing. Excessive inflammation can lead to tissue damage and delayed healing, while excessive suppression of inflammation can hinder normal cellular function [6]. Hence, precise control and modulation of the inflammatory response are critical steps in wound repair [7,8]. Wound infection is also a common challenge in wound repair. Wounds provide a gateway for bacteria and other microorganisms to enter the body, making them susceptible to invasion [1]. If infections are not promptly and effectively controlled, they can have a significant impact on wound recovery. Infections may lead to worsening of the wound, prolong the healing process, and increase the risk of complications [9]. Therefore, a key aspect of wound management involves appropriate infection prevention and treatment measures, including wound cleansing, antibiotic use, and regular monitoring [10]. Furthermore, angiogenesis, the formation of new blood vessels, plays a crucial role in wound repair [11]. The development of new blood vessels provides damaged tissues with oxygen and nutrients, promoting the growth and repair of new tissue [12]. This process is highly complex and finely regulated, and is controlled by various growth factors and cytokines. Certain chronic diseases, such as diabetes, can disrupt angiogenesis, thereby affecting wound healing [13]. Therefore, wound repair is a multifaceted process influenced by various interacting factors. In the treatment of wound healing, it is essential to consider factors such as tissue inflammation, wound infection, and angiogenesis, all of which play vital roles in the overall wound repair process [14].

Extracellular vesicles (EVs) are a class of small lipid vesicles that are widely present in various cell types and have been found to contain a variety of bioactive components [15,16]. These bioactive components include proteins, nucleic acids, lipids, and cytokines, which can regulate cellular physiological and metabolic processes and promote the healing of injured sites [17,18,19]. They not only carry growth factors and cytokines that stimulate the regeneration of damaged tissues but also regulate inflammatory responses, reducing inflammation caused by injuries [20,21]. Furthermore, extracellular vesicles can promote the formation of new blood vessels, improving the blood supply to wounds, thus aiding in the healing and repair of wounds [12,22]. This has provided new breakthroughs and hope in the field of wound repair. However, directly applying EVs to a wound can lead to their diffusion through bodily fluids, resulting in a suboptimal concentration at the intended wound site [23,24,25,26]. This can result in reduced efficacy and a shorter therapeutic duration. To maintain an optimal dose, the medication needs to be applied continuously, making the procedure more complex and potentially decreasing patient adherence [21]. Hence, there remains a pressing need for a more effective and long-lasting approach to enhance wound healing.

In this study, we present a programmable strategy designed to optimize the wound microenvironment and accelerate healing. We engineered a bioactive hydrogel embedded with extracellular vesicles (EVs) derived from anti-inflammatory M2 macrophages and loaded with zeolitic imidazolate framework-8 (ZIF), an antibacterial nanomaterial. The resulting ZIF@EVs were incorporated into a sodium alginate matrix to form an EV-infused hydrogel (“EV–Gel ink”). This bioactive hydrogel is designed for direct application to chronic wounds, providing sustained release of the therapeutic EVs. Our results demonstrate that this EV–Gel ink promotes endothelial cell proliferation and migration, facilitating angiogenesis, and effectively reprograms inflammatory macrophages toward the M2 phenotype, shifting the wound’s immune environment from inflammation to a more regenerative state. The therapeutic potential of this EV-infused hydrogel was validated in vivo using a murine chronic wound model, where it significantly accelerated healing compared to conventional treatments. This study offers a novel, dual-action approach that not only enhances tissue repair but also modulates the immune microenvironment for improved chronic wound management.

## 2. Results

**Figure 1:**
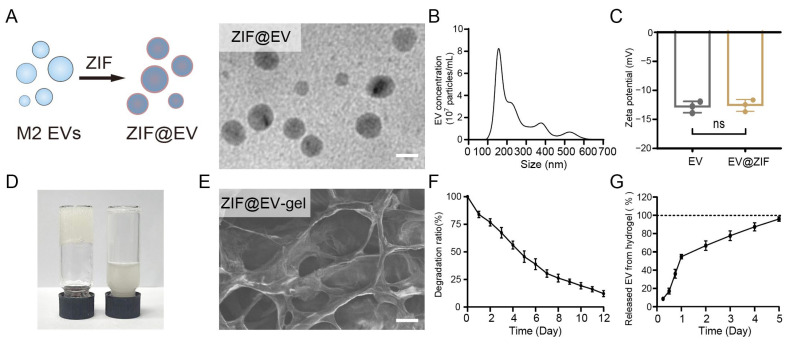
Synthesis of ZIF@EV–Gel ink. (**A**) Schematic illustration of the synthetic process of ZIF@EV nanoparticles and corresponding TEM images of the prepared ZIF@EV. Scale bar: 100 nm. (**B**) NTA of the prepared ZIF@EVs demonstrating the average size of around 180 nm. (**C**) Zeta potential of EV and ZIF@EV. (**D**) The gelation of ZIF@EV–Gel ink. (**E**) SEM image of the macrostructure of ZIF@EV–Gel ink. Scale bar: 3 μm. (**F**) The degradation curve of ZIF@EV forms the Gel. (**G**) The continuous release profile of ZIF@EV from Gel. ns denotes non-significant difference.

Chronic wounds are typically in an inflammatory state, which impedes efficient tissue healing. We initiated our study by preparing ZIF@EV nanostructures to explore their potential biomedical applications. As depicted in Figure 1A, the encapsulation process involved incorporating ZIF-8 into M2 extracellular vesicles (EVs) to form ZIF@EV. The ZIF nanoparticles were first prepared for EV loading (Figure S1). This process is visually represented through a schematic diagram and corroborated by transmission electron microscopy (TEM) images, which depict spherical ZIF@EV particles. In the final product ZIF@EVs, the embedding efficiency of ZIF-8 was about 50%, with the recovery efficiency of EVs determined to be about 70%. Subsequent nanoparticle tracking analysis (NTA) revealed that the diameter of ZIF@EV particles falls within the 100 nm to 400 nm range, aligning with the size distribution of the original EVs, as shown in Figure 1B [27,28]. Zeta potential measurements demonstrated that both EV and ZIF@EV are negatively charged, and importantly, the electric potential of EVs remains unaffected by the incorporation of ZIF-8, indicating stability in their surface charge properties (Figure 1C).

To further investigate the practical applications of these nanostructures, we mixed EVs with a sodium alginate precursor solution to form a hydrogel, referred to as ZIF@EV–Gel ink, within 3 min (Figure 1D). Scanning electron microscopy (SEM) analysis revealed that the ZIF@EV–Gel ink maintains a distinct mesh-like structure, with no significant alterations observed before and after the loading of ZIF@EV into the hydrogel (Figure 1E). This structural consistency is crucial for maintaining the integrity and functionality of the hydrogel in potential applications. Figure 1F shows a gradual decrease in the hydrogel’s mass, indicating that the material undergoes controlled degradation. To analyze the degradation profile and EV release from the hydrogel, five kinetic models (zero-order kinetics, first-order kinetics, the Higuchi model, the Hixon–Crowell model, and the Korsmeyer–Peppas model) were applied [29,30,31,32,33]. The zero-order model describes systems where the release rate is constant; this is commonly applicable to transdermal patches or matrix tablets. The first-order kinetics model explains a system where the release rate is proportional to the remaining drug concentration, typically used for oral dosage forms and other pharmaceutical preparations. The Higuchi model is based on diffusion-controlled mechanisms, often used for planar systems and matrix-type drug delivery systems. The Hixon–Crowell model accounts for systems where the surface area of the drug particle changes during degradation or dissolution. The Korsmeyer–Peppas model is a semi-empirical equation widely used for polymeric systems. The analysis of the data using kinetic models revealed that the first-order model best described the degradation profile of hydrogel (Figure S2), as evidenced by the highest R2 value (0.995), revealing that the degradation rate of hydrogel is proportional to the remaining hydrogel. Additionally, the Higuchi model with an R^2^ value of 0.943 (Figure 1G), demonstrated that the release of encapsulated EVs was primarily governed by diffusion-driven mechanisms (Figure S3), with the encapsulated vesicles being fully released by day 5. These findings align with the sustained therapeutic delivery goals of hydrogels, demonstrating controlled degradation and prolonged EV release with minimal potential side effects, making these hydrogels suitable for various biomedical applications. Overall, our findings suggest that ZIF@EV nanostructures, when incorporated into a hydrogel matrix, exhibit promising characteristics for biomedical applications, including stability, controlled degradation, and effective release of therapeutic agents, thereby further enhancing their cutaneous microenvironment modulation capability for wound healing.

**Figure 2:**
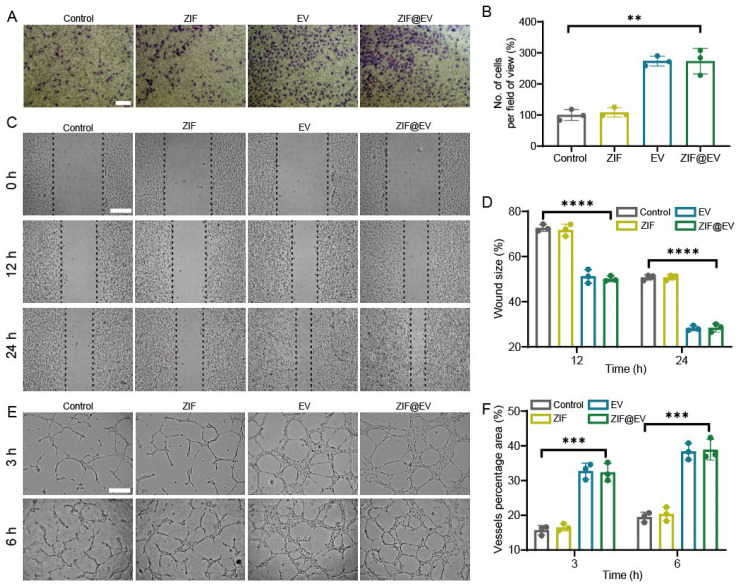
Angiogenesis ability of ZIF@EV. (**A**) Representative images of the transwell migration assay of HUVECs under the treatment of ZIF@EV. Scale bar, 200 μm. (**B**) Quantification of the migratory capacity of HUVECs. (**C**) Images of HUVEC migration under ZIF@EV treatment at different time points. Scale bar, 200 μm. (**D**) Corresponding quantification of HUVEC migration at different time points. (**E**) Formation of tubes by HUVECs with various treatments. (**F**) percentage area of vessels as a representation of tube formation capability in various groups (n = 3). ** *p* < 0.01, *** *p* < 0.001, and **** *p* < 0.0001.

The differentiation of vascular endothelial cells plays a pivotal role in the process of wound healing. During the process of vascular regeneration, intracellular cytokines stimulate endothelial cell division, proliferation, migration, and the formation of new blood vessels [34,35]. Therefore, the proliferation and migration of vascular endothelial cells are vital for vascular regeneration. Cell toxicity assay demonstrated that ZIF@EV significantly promoted the proliferation of HUVECs. In the transwell assay, ZIF@EV was introduced to the upper chamber and co-cultured with HUVECs for a duration of 24 h. In comparison to the control group, ZIF@EV enhanced the migration ability of HUVECs (Figure 2A,B). Furthermore, a scratch assay of HUVEC migration revealed that the cell migration rate of HUVECs in the EV-treated group was significantly higher than that in the control group at 12 h and 24 h, with a concentration-dependent effect (Figure 2C,D). Additionally, Tube formation experiments were conducted to investigate the impact of EV on endothelial cell differentiation [36]. The results showed that cells treated with ZIF@EV exhibited more capillary formation compared to the untreated control group. Vascular regeneration showed time dependency, with 6 h of vascular regeneration being more significant than 3 h (Figure 2E). After treatment with ZIF@EV at different concentrations, the total length of blood vessels, the percentage of vascular area, and the total number of vascular nodes all significantly increased in a concentration-dependent manner. Overall, ZIF@EV can stimulate endothelial cell proliferation and migration, thereby boosting angiogenic potential.

**Figure 3:**
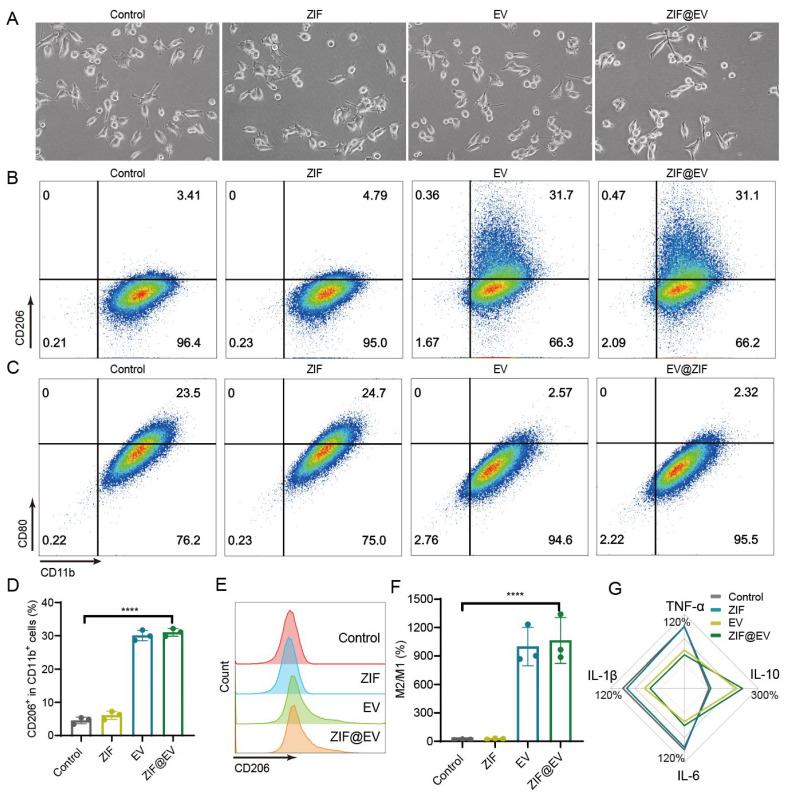
ZIF@EV induced macrophage polarization into M2 phenotype. (**A**) Representative image of macrophages under different treatments. Scale bar, 50 μm. (**B**) Flow cytometry analysis of CD206+ macrophages. (**C**) Flow cytometry analysis of CD86+ macrophages. (**D**) Quantification of CD206+ macrophages (n = 3). (**E**) Histogram analysis of CD206+ macrophages. (**F**) Quantification of the ratio of M1 and M2 macrophages (n = 3). (**G**) Relative protein expression of M1 and M2 macrophage markers after macrophages incubated with various treatments (n = 3). The color contour from blue to red denotes the intensified signal. **** *p* < 0.0001.

Macrophages, a type of white blood cell, are vital for the body’s defense against foreign invaders and play a crucial role in immune response and tissue repair. To evaluate the efficacy of ZIF@EV in promoting macrophage polarization into the M2 phenotype, we conducted an in vitro study designed to observe both morphological and functional changes in macrophages. Initially, M0 macrophages were exposed to lipopolysaccharide (LPS) for 24 h to induce an inflammatory response, mimicking the conditions necessary for subsequent polarization. Following this treatment, the macrophages were co-cultured with ZIF@EV. Compared to the control group, we observed a notable reduction in macrophages with short pseudopods and a round shape, while a significant number exhibited elongated pseudopods typical of the M2 phenotype (Figure 3A). This morphological transformation indicates a shift in macrophage phenotype from the pro-inflammatory M1 to the anti-inflammatory M2. To confirm this phenotypic shift, we employed flow cytometry analysis to detect specific surface markers associated with macrophage polarization. We used CD206 as a marker for M2 macrophages and CD86 for M1 macrophages (Figure 3B,C). The flow cytometry results demonstrated that ZIF@EV treatment significantly increased the expression of CD206 and decreased the expression of CD86, thus confirming the repolarization of macrophages towards the M2 phenotype (Figure 3D,E). This was further evidenced by a substantial increase in the M2 to M1 macrophage ratio in the ZIF@EV-treated group compared to the control group (Figure 3F).

To explore the underlying mechanisms of ZIF@EV-induced macrophage polarization, we conducted an ELISA assay to measure the levels of inflammatory cytokines in the cell supernatant. The results showed that ZIF@EV significantly elevated the levels of the anti-inflammatory cytokine IL-10, while concurrently reducing the levels of pro-inflammatory cytokines TNF-α, IL-1β, and IL-6 (Figure 3G). These findings suggest that ZIF@EV not only induces morphological and phenotypical changes indicative of M2 polarization but also modulates the inflammatory environment to favor an anti-inflammatory response. This dual action of ZIF@EV—promoting M2 polarization and modulating cytokine expression—highlights its therapeutic potential. By facilitating tissue repair and enhancing wound healing through macrophage modulation, ZIF@EV could serve as a promising candidate for advanced therapeutic applications. The ability to shift macrophages from an inflammatory to a regenerative state positions ZIF@EV as a valuable tool in the development of treatments aimed at improving recovery outcomes in various inflammatory and injury-related conditions.

**Figure 4:**
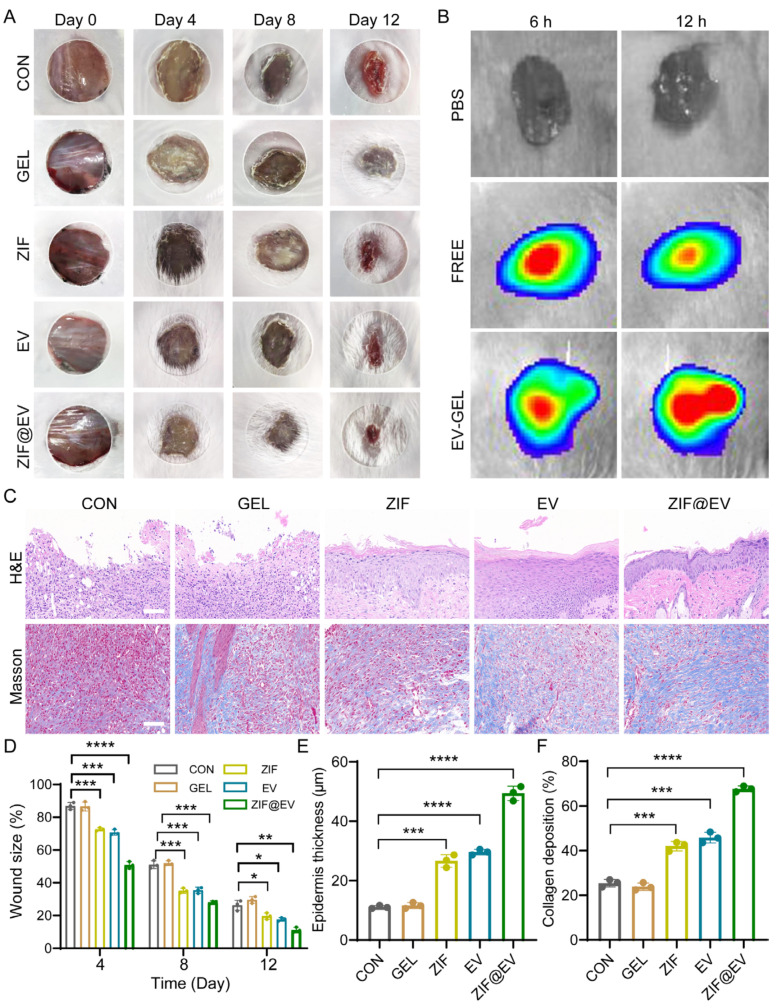
In vivo wound healing efficacy of ZIF@EV-Gel. (**A**) Representative image of wound size on different days. (**B**) Fluorescence images of cutaneous wounds extracted at 6 and 12 h after different treatments. The color contour from blue to red denotes the intensified signal. (**C**) Hematoxylin and eosin (HE) and Masson stain of the wounded skin on day 12. Scale bar = 100 μm. (**D**) Quantification of wound size in different groups. (**E**) Quantitative analysis of epidermal thickness. (**F**) Quantitative analysis of collagen deposition (n = 3). * *p* < 0.05, ** *p* < 0.01, *** *p* < 0.001, and **** *p* < 0.0001.

To evaluate the therapeutic efficacy of ZIF@EV-Gel ink on wound healing, we established a full-thickness mouse wound model. The mice were randomly divided into five groups: Control, Gel, ZIF, EV, and ZIF@EV. Treatments were applied to the wound surface on days 0, 4, 8, and 12, with photographs taken at each interval to document wound area reduction. The ZIF@EV-Gel ink group demonstrated significantly enhanced wound healing compared to the control group (Figure 4A). Both the ZIF and EV groups showed effective wound healing promotion; however, the combination of EV and ZIF in the ZIF@EV-Gel ink resulted in even faster healing, indicating superior therapeutic efficacy (Figure 4D). For further analysis, EVs were labeled with DID, and both free EV and ZIF@EV-Gel ink were applied to the wound surfaces in vivo. Observations at 6 and 12 h post-application revealed the presence of EVs on the tissue, indicating that EV-Gel gradually released extracellular vesicles at the wound site, ensuring a sustained supply of EVs over time (Figure 4B).

During wound healing, epidermal thickness increases during the proliferative and inflammatory stages and decreases during the remodeling stage. Epidermal thickness is a critical factor in tissue remodeling. Therefore, on the 12th day of treatment, we performed histological evaluation of the wound using H&E staining (Figure 4C). Quantitative analysis showed that ZIF@EV-Gel ink significantly increased the thickness of the epidermis (Figure 4C,E). Additionally, on the 12th day of treatment, we assessed collagen deposition at the wound site using Masson’s staining (Figure 4C). Quantitative analysis revealed that wounds treated with EV-Gel ink exhibited denser and more organized collagen fiber deposition (Figure 4F). These findings underscore the enhanced therapeutic efficacy of ZIF@EV-Gel ink in promoting wound healing and tissue regeneration through sustained EV release and improved collagen deposition.

**Figure 5:**
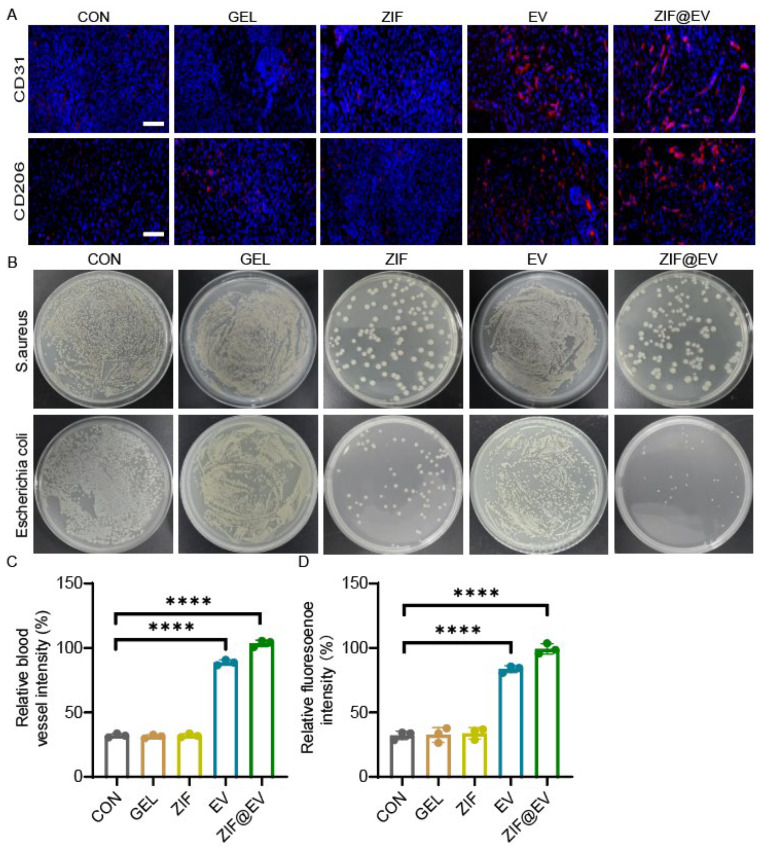
Therapeutic mechanism of ZIF@EV-Gel ink. ZIF@EV-Gel accelerated wound healing in vivo. (**A**) Image of immunofluorescence staining to assess the formation of new blood vessels and the inflammatory status of the wounds. The blue color indicate nucleus and red color indicate the presence of CD31 and CD206 separately. (**B**) bactericidal effects of ZIF@EV-Gel. (**C**) Quantification of blood vessels with various treatments. (**D**) Quantitative analysis of activated M2 macrophages in epidermal tissues. **** *p* < 0.0001.

To elucidate the therapeutic mechanism of ZIF@EV-Gel ink and assess its effects on vascular regeneration and macrophage phenotypes, we conducted immunofluorescence staining for CD31 and CD206. This staining enabled us to evaluate new blood vessel formation and the inflammatory status of wounds after 12 days of treatment (Figure 5A). Quantitative analysis revealed a significant impact of ZIF@EV-Gel ink on wound healing. Notably, the application of ZIF@EV-Gel ink significantly increased the density of microvessels at the wound site, enhancing blood supply and nutrient delivery crucial for the healing process. Moreover, the ink activated macrophages towards the M2 phenotype, associated with anti-inflammatory effects, as evidenced by increased CD206 expression. This polarization reduces tissue damage and promotes tissue repair (Figure 5C,D). To further investigate the bactericidal effects of the different treatment groups, we treated Staphylococcus aureus and Escherichia coli with the agents. The results indicated that both the ZIF group and the ZIF@EV-Gel group effectively killed bacteria and inhibited bacterial growth at the wound site, demonstrating strong antibacterial properties (Figure 5B). These findings indicate that ZIF@EV-Gel ink not only promotes vascular regeneration by increasing microvessel density but also induces macrophage polarization towards the M2 phenotype, modulating the inflammatory environment to favor healing. Additionally, its antibacterial effects contribute to a more sterile wound environment, further facilitating recovery. The in vivo ability of ZIF@EV-Gel to promote vascular regeneration, enhance microvessel density, induce beneficial macrophage polarization, modulate inflammation, and exert antibacterial effects aligns with its promising in vitro results. Collectively, these properties position ZIF@EV-Gel ink as a potent therapeutic agent that accelerates wound healing through multiple synergistic mechanisms.

## 3. Materials and Methods

### 3.1. Materials

Sodium alginate purchased from Sinopharm Chemical Reagent Co., Ltd. (Shanghai, China). Calcium carbonate (CaCo_3_) purchased from Xilong Scientific Co., Ltd. (Guangdong, China). D-(+)-Gluconic acidδ-lactone (99%) purchased from Aladdin (Shanghai, China).

### 3.2. Preparation of EVs from M2 Macrophages

M2 macrophages were cultured, and the conditioned medium was collected for extracellular vesicle (EV) isolation. The medium was first centrifuged at 500× *g* to remove cells and debris, followed by a second centrifugation at 10,000× *g* for 90 min to remove larger vesicles. The supernatant was then collected and subjected to ultracentrifugation at 100,000× *g* for 2 h to pellet the EVs. The resulting precipitate was resuspended in phosphate-buffered saline (PBS) and stored at −80 °C for subsequent use.

### 3.3. Preparation of ZIF-Loaded EV Nanoparticle

For the synthesis of ZIF nanoparticles, a solution of 20 mg of Zn(NO_3_)_2_·6H_2_O and 0.38 g of HmIm was prepared by dissolving the compounds in 2.5 mL of dH_2_O. After stirring at room temperature for 10 min, a white emulsion formed. The mixture was then centrifuged at 12,000 rpm for 15 min to collect the ZIF precipitate, which was washed three times with dH_2_O. The ZIF nanoparticles were finally dispersed in water for further usage. For the preparation of ZIF@EV nanoparticles, a total of 10 μg of purified M2-EVs was combined with 50 μg of ZIF nanoparticles, and the resulting mixture underwent electroporation in a 4mm electroporation cuvette (X-Porator H1) under conditions of 1000 kV for 5 ms. This electroporation facilitated the puncturing of M2-EVs, creating microscopic holes to ensure efficient loading of ZIF nanoparticles. Following electroporation, the medium containing the ZIF@EVs was subjected to two washes with phosphate-buffered saline (PBS) to remove any unloaded ZIF nanoparticles. The resultant product, ZIF@EV, was then incubated at 37 °C for 3 h. This incubation step allowed for potential interactions and stabilization of the loaded ZIF within the M2-EVs. The final purified ZIF@EV product was resuspended in PBS, making it suitable for further experimental applications.

### 3.4. Preparation and Characterization of ZIF@EV-Gel

The ZIF@EV-Gel ink was formed by combining sodium alginate, calcium carbonate, D-(+)-gluconic acid δ-lactone, and extracellular vesicles (EVs) obtained from M2 macrophages. Initially, a solution containing 2% sodium alginate and calcium carbonate was mixed for 30 min until complete fusion occurred. Following this, EVs and gluconolactone were added. The resulting mixture of ZIF@EV, gluconolactone, and SA@CaCO_3_ was gently magnetically stirred at 50 g for 3 min at 26 °C to form ZIF@EV-Gel.

### 3.5. Characterization of ZIF@EVs

ZIF@EVs intended for TEM observation were obtained through ultracentrifugation (100,000× *g* for 2 h) and later suspended in PBS. A droplet containing EVs was placed onto a glass slide, and a copper mesh was positioned over the droplet for 10 min. Following this, removing the copper mesh and dry up any surface water with filter paper. The sample was then fixed with glutaraldehyde for 5 min and then washed with PBS. Subsequently, 10 μL of a 1% solution of uranium acetate was applied for negative staining. The sample was then cleaned with PBS, allowed to air-dry, and finally subjected to imaging using a transmission electron microscope (Hitachi, HT7700, Tokyo, Japan). Particle sizes were determined by nanoparticle tracking analysis (NTA, Particle Metrix Zetaview, PMX 110, Isny, Germany). The zeta potential were measured by ZetaPALS (Brookhaven, v7.03, New York, NY, USA).

### 3.6. Assessment of Macrophage Polarization

To study ZIF@EV’s effect on macrophage polarization, various ZIF@EV formulations were used in macrophage culture. In brief, A total of 10,000 macrophages were carefully placed in each well of a 6-well plate. The plate was then filled with DMEM containing 10% FBS, and the macrophages were allowed to grow and develop overnight. Subsequently, PBS and ZIF@EV were introduced to each well, and the cells were cultured for 48 h. Optical microscopy was utilized to document the morphological alterations of the macrophages. After a 48-h treatment with PBS and ZIF@EV, a suspension of macrophages was prepared and cultured with anti-mouse CD206 (0.5 μg/mL), anti-mouse CD11b (0.4 μg/mL), and anti-mouse CD80 (0.25 μg/mL) at 37 °C for 30 min in the dark. The cell phenotype was then analyzed by flow cytometry using BECKMAN COULTER Cyto-FLEX and the results were analyzed using Flow cytometry (Agilent NovoCyte, Aminis Flowsight, Santa Clara, CA, USA) with Flow software (FlowJo v10.8.1).

### 3.7. Cytokine Analysis

The concentration of inflammatory cytokines was measured following the manufacturer’s instructions using an ELISA kit (4A Biotech Co. Ltd., Beijing, China). Macrophages were incubated with ZIF, EVs, or ZIF@EVs for 24 h, after which the supernatant was collected. The levels of macrophage-related cytokines, including anti-inflammatory marker IL-10 and pro-inflammatory markers TNF-α, IL-1β, and IL-6, were then assessed.

### 3.8. Cell Scratch Assay

The migration of HUVECs was assessed using the cell scratch assay. Human umbilical vein endothelial cells (HUVECs) were seeded in 6-well plates and cultured for a period of 24 h. Subsequently, a sterile pipette tip was employed to create a wound, and PBS was used for washing the cells in order to eliminate any unattached cells. The attached cells were then cultured in serum-free extracellular matrix and treated for varying durations of 0, 12, and 24 h under diverse conditions. Finally, cell images were captured with an optical microscope (magnification of 40×) and quantification of the cell migration rate was performed using ImageJ software (1.8.0). The migration rate was determined by calculating the ratio of the healed wound to the initial wound, multiplied by 100%.

### 3.9. Tube Formation Assay

To explore the effects of ZIF@EV on angiogenesis, we utilized the tube formation assay. The process began with pre-cooling the pipet tip and 96-well plate, followed by plating Matrigel per well in the 96-well plate on ice. Afterward, the plate was moved to a 37 °C incubator for one hour to allow for gel formation. Next, HUVECs (1 × 10^4^ cells/well) were seeded into the 96-well plate and treated with both PBS and ZIF@EV. Subsequent to varying incubation periods, tube formation images were captured using an optical microscope, followed by analysis using the ImageJ software. The quantitative metrics obtained included the aggregate length of vessels, the extent of vascular coverage in the sample, and the total count of junctions.

### 3.10. In Vivo Wound Healing Evaluation

The Balb/C mice were subjected to isoflurane anesthesia, and their dorsal hair was shaved before creating full-thickness skin wounds of approximately 9–10 mm diameter. The study employed five groups: Saline (Control), Gel, ZIF, EV, and ZIF@EV-Gel group, and the mice were randomly assigned to each group, with three mice in each group. The hydrogel was applied on days 0, 4, 8, and 12, and wound areas were photographed and calculated using ImageJ. After 12 days of treatment, the mice were euthanized, and wound tissues were collected for subsequent histopathological and immune analyses. To evaluate the retention of ZIF@EV from the embedded hydrogel on the wounds and its interaction with host cells, Free ZIF@EV and ZIF@EV-Gel were administered to the wounds. H&E and Masson staining were employed to evaluate epidermal thickness and collagen deposition in wounds after the 12-day treatment period. The wound tissues were fixed with 4% paraformaldehyde, paraffin-embedded after dehydration, and then sectioned into 5 μm thick slices for H&E and Masson staining. Furthermore, immunofluorescence staining was conducted to assess the expression of CD206 and CD31 in wounds after the 12-day treatment, with the images captured using a microscope.

### 3.11. Sterilization Characteristic Evaluation

The wound tissues collected from the first 5 groups of Balb/C mice will be ground and filtered under sterile conditions. The filtered bacterial solution will be diluted 10^8^ times, and then 100 uL of each diluted bacterial solution will be evenly inoculated onto the culture medium. After 24 h, photos will be taken for observation and the colony count will be counted.

## 4. Statistical Analysis

Statistical analysis was performed using GraphPad Prism software (7.01), employing one-way analysis of variance (ANOVA). Data are presented as means ± standard deviations. Differences were considered statistically significant at * *p* < 0.05, ** *p* < 0.01, *** *p* < 0.001, and **** *p* < 0.0001.

## 5. Conclusions

In this study, we have developed an therapeutic EV patch to enhance wound healing. This biologically active agent, derived from M2-macrophage cells loaded with ZIF, creates bioactive ZIF@EVs. By incorporating these vesicles, which contain antibacterial agents, into a sodium alginate hydrogel, we formed a rapidly deployable and biologically reparative patch. 

The specially designed patch promotes the growth and migration of endothelial cells, significantly enhancing angiogenesis potential. Additionally, it repolarizes inflammatory macrophages towards the M2 phenotype, shifting the immune microenvironment from inflammatory to proliferative. However, while our in vitro migration assays for human umbilical vein endothelial cells (HUVECs) did not show a significant difference between treatments with EV and ZIF@EV-Gel, the in vivo wound healing results were markedly improved with the use of ZIF@EV-Gel ink. This apparent discrepancy can be attributed to therapeutic mechanisms that are more prominent in the in vivo environment, particularly the antibacterial effects of ZIF. We observed that both the ZIF and ZIF@EV-Gel treatments demonstrated strong bactericidal activity, effectively eliminating *Staphylococcus aureus* and *Escherichia coli* at the wound site (Figure 5B). The antibacterial property of ZIF is especially critical in the context of chronic wounds, which are often susceptible to bacterial infections that hinder the healing process. By reducing the bacterial load and maintaining a sterile wound environment, ZIF creates favorable conditions for tissue repair and regeneration. This antibacterial action is not captured in the in vitro HUVEC migration assay, where bacterial presence is absent.

Therefore, the regenerative capabilities of this bioactive hydrogel patch were further validated using a mouse model, underscoring its promise as a portable and effective bio-reparative platform for wound healing. The in vivo results highlight the broader therapeutic potential of ZIF@EV-Gel, which not only promotes angiogenesis and modulates the immune response but also provides essential antibacterial effects crucial for managing chronic wound infections. This multifaceted approach underscores the importance of considering the complex in vivo environment when evaluating the efficacy of wound healing treatments.

## Data Availability

All data were present in the main text.

## Supplementary Materials

The following supporting information can be downloaded at: https://www.mdpi.com/article/10.3390/ijms252313093/s1.
