# Supplementary material for: Bioengineered Extracellular Vesicle Hydrogel Modulating Inflammatory Microenvironment for Wound Management
Source: Int J Mol Sci. 2024 Dec 5;25(23):13093. doi: 10.3390/ijms252313093 (PMC11642447; doi:10.3390/ijms252313093)
Supplement: Supplementary file 1 [file ijms-25-13093-s001.zip › ijms-3315606-supplementary.pdf]

Supplementary Materials

Bioengineered Extracellular Vesicle Hydrogel Modulating Inflammatory Microenvironment for Wound Management

Yunfei Mu [1], Liwen Ma [2], Jia Yao [1], Dan Luo [2,*] and Xianguang Ding [1,*]

[1] State Key Laboratory of Organic Electronics and Information Displays, Jiangsu Key Laboratory of Smart Biomaterials and Theranostic Technology, Nanjing University of Posts and Telecommunications, Nanjing 210023, China; 1223066206@njupt.edu.cn (Y.M.); 1023233416@njupt.edu.cn (J.Y.)

[2] The First College of Clinical Medicine, Nanjing Medical University, Nanjing 211100 China

* Correspondence: daniluo2013@njmu.edu.cn (D.L.); iamxgding@njupt.edu.cn (X.D.)

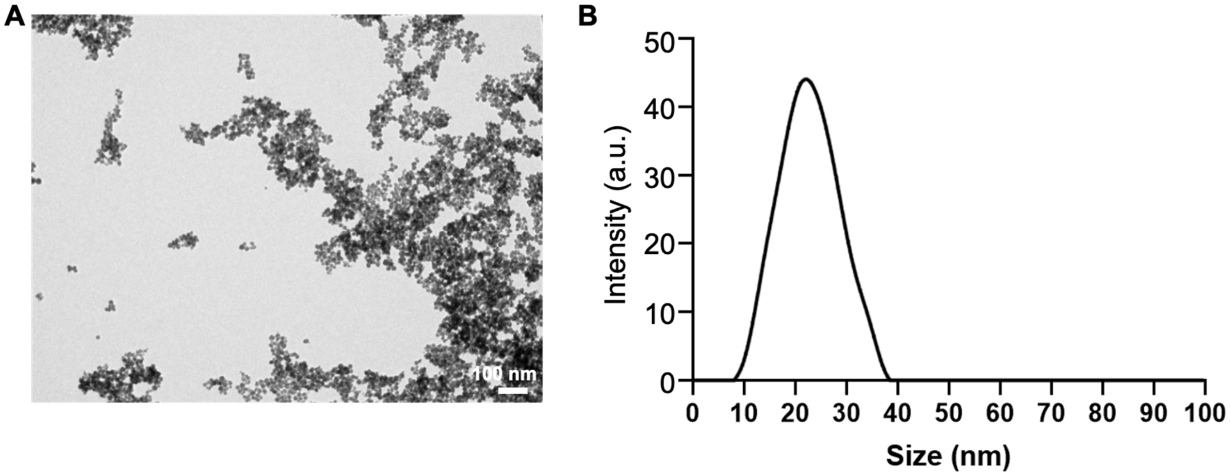

Figure S1. (**A**) TEM image of prepared ZIF nanoparticles. (**B**) Hydrodynamic size of ZIF particles measured by DLS.

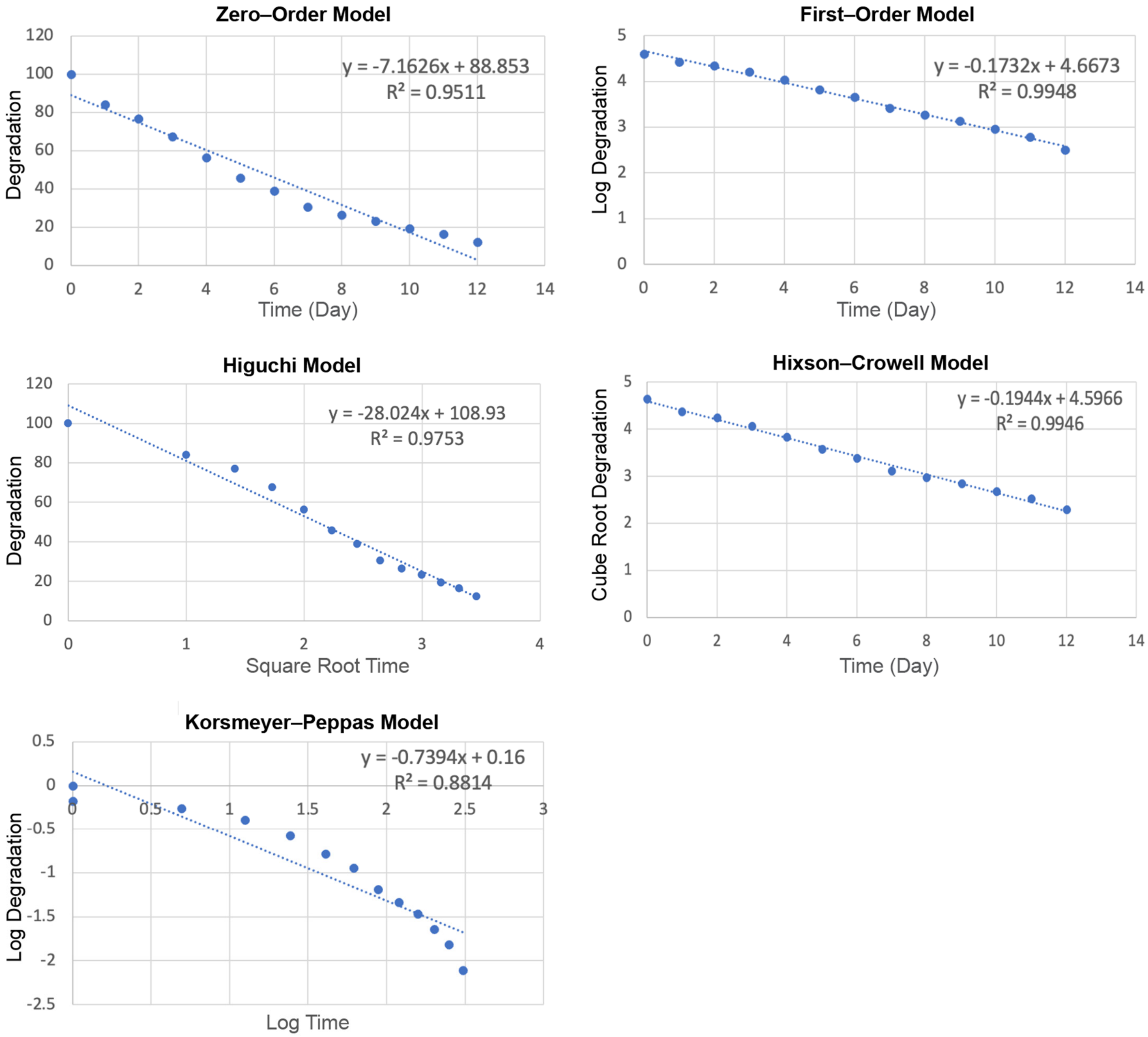

Figure S2. Mathematical models used for linear regressions of the degradation profile of the Gel.

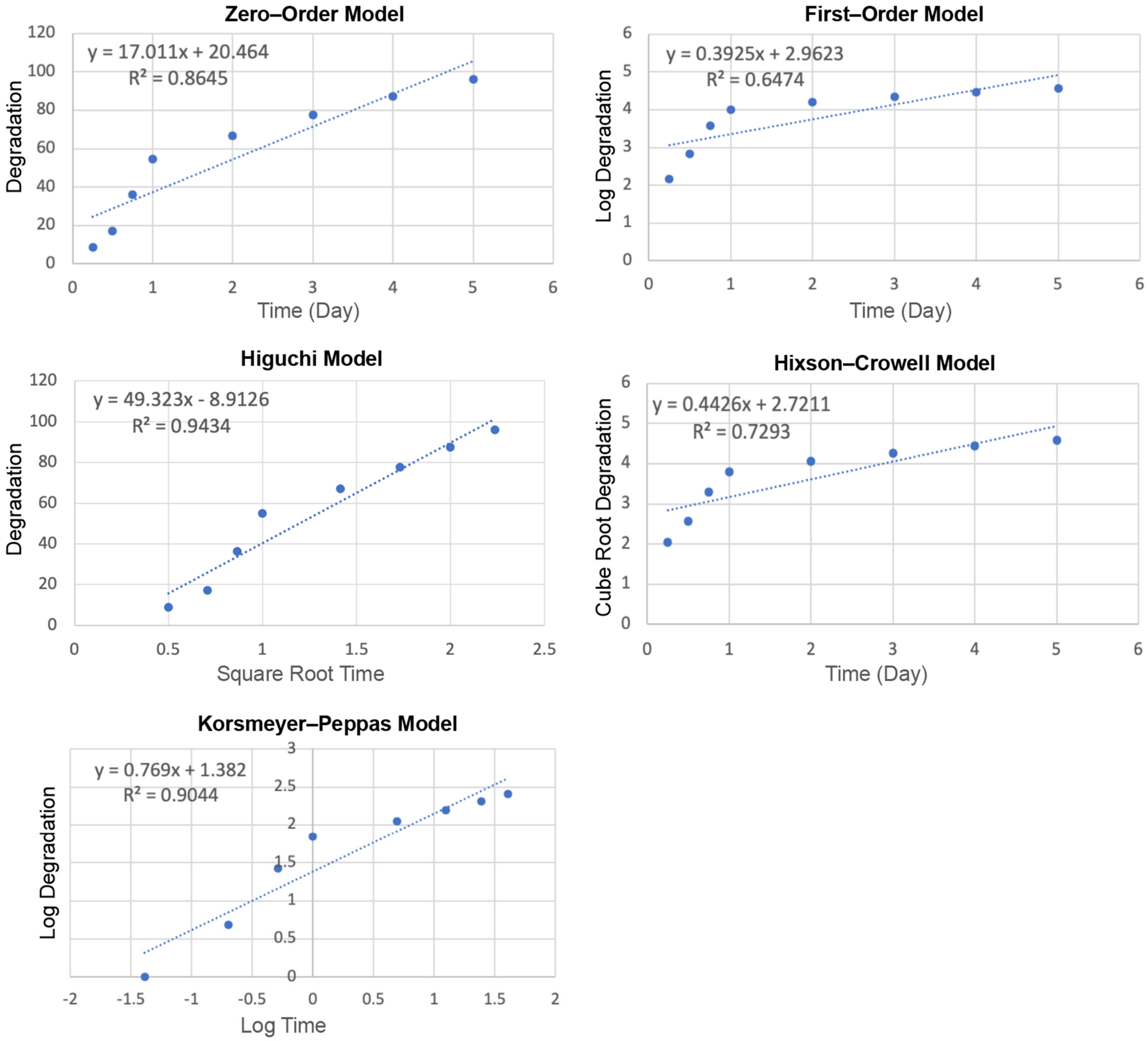

Figure S3. Mathematical models used for linear regressions of the continuous release profile of ZIF@EV from Gel.
